# Ischiopubic Osteochondrosis (Van Neck-Odelberg Disease) as a Rare Cause of Pediatric Hip Pain: A Systematic Review

**DOI:** 10.7759/cureus.85846

**Published:** 2025-06-12

**Authors:** Cameron Gerhold, Renish N Contractor, Matthew McGee, Jacob Myhre, Michael Sweeney

**Affiliations:** 1 Orthopedic Surgery, Florida State University College of Medicine, Tallahassee, USA; 2 Urology, Florida State University College of Medicine, Daytona Beach, USA; 3 College of Medicine, Lake Erie College of Osteopathic Medicine, Bradenton, USA; 4 College of Medicine, Florida State University College of Medicine, Tallahassee, USA

**Keywords:** musculoskeletal imaging, non-surgical orthopedics, orthopedics & traumatology, pediatric hip pain, pediatric orthopedic surgery

## Abstract

Overgrowth of the ischiopubic synchondrosis, a junction between the inferior pubic ramus and ischium, leads to the development of Van Neck-Odelberg disease. This condition is a rare cause of pediatric hip pain and limping that is often misdiagnosed due to its rarity and non-specific presentation. A review of the literature was conducted on PubMed, Cochrane, Embase, and Web of Science to identify case reports and case series of Van Neck-Odelberg disease using the keywords “Van Neck-Odelberg Disease” and “Ischiopubic Osteochondrosis.” A total of 115 studies were identified. After applying exclusion criteria, 15 studies remained. Most patients were male (n = 16; 84.2%), and the median age was 12 years (range = 5.5-17 years). The most common presenting symptoms were groin pain (n = 16; 84.2%), limping and gait limitation (n = 9; 47.4%), and restricted hip range of motion (n = 4; 21.1%). The most common differential diagnoses were fractures (n = 7; 36.8%), ischial ramus tumors (n = 6; 31.6%), and osteomyelitis (n = 5; 26.3%). The median time to symptom resolution was eight weeks, with most patients achieving complete resolution of symptoms. The major challenge with this condition is preventing misdiagnosis of pediatric patients. The most common misdiagnosis was a fracture. Understanding the anatomy of physiology of the pediatric hip and raising awareness of this rare condition will likely benefit physicians struggling to make this diagnosis. Recognizing common presenting signs of Van Neck-Odelberg disease and diagnostic imaging features will allow physicians to make the correct initial diagnosis, potentially preventing invasive treatments in pediatric patients.

## Introduction and background

Van Neck-Odelberg disease (VND) is a skeletal abnormality consisting of an overgrowth of the ischiopubic synchondrosis (IPS), which serves as the junction between the inferior pubic ramus and ischium [1]. The condition was termed Van Neck-Odelberg after it was first described in the early 1920s by Odelberg and Van Neck and was described as “osteochondritis ischiopubica” [2,3]. VND is a rare condition that typically occurs in adolescents during skeletal maturation and can occur unilaterally or bilaterally. Patients typically present with groin or gluteal pain and difficulty ambulating.

The ischiopubic area of the pelvis is formed by the superomedial aspect of the pubis and the posterolateral aspect of the ischium. There is cartilaginous tissue that forms a joint where these two bones meet, called the IPS, and an overgrowth of this tissue causes VND [4]. This area forms embryologically at five to six months of fetal life, and fusion of the cartilage is complete at term. Ossification of the cartilage occurs during puberty. In rare instances, asymmetrical growth of the IPS can lead to increased stress on the pelvis, leading to VND [5]. This leads to an inflammatory response, which can subsequently delay ossification of the IPS [6].

The condition is self-limiting and can occur until ossification of the IPS occurs [7]. Once ossification occurs during puberty, no residual deformity is found. This condition can mimic fracture, infection such as osteomyelitis, trauma, tumors, or enthesitis-related arthritis. Clinically, pain in the pelvic region with the absence of fever and normal laboratory results helps narrow down the differential diagnosis. We present a systematic review of all published reports of VND, including details on presenting symptoms, diagnostic imaging results, treatments, and other details about the cases. The purpose of this review is to provide the most comprehensive overview of this rare disease with the aim of determining if there is an optimal treatment option.

## Review

Methodology

This systematic review on VND adhered to the Preferred Reporting Items for Systematic Reviews and Meta-Analyses (PRISMA) checklist [8]. Additionally, it was registered on PROSPERO (ID: 1040621). This review aims to review the various presentations of VND and determine is there is an optimal treatment option for this rare diagnosis.

Eligibility Criteria

This review included all studies that were either a case report or a case series on VND. Studies were excluded if (1) VND was not the final diagnosis, (2) important details regarding diagnostic workup and treatment were not included, (3) they were designed as cross-sectional, cohort, case-control, and clinical trials; or (4) meta-analyses or systematic reviews.

Search Strategy

A literature search was conducted on PubMed, Cochrane, Embase, and Web of Science to identify case reports and case series of VND, also known as ischiopubic osteochondrosis, up to October 1, 2023. Keywords utilized in the database searches were “Van Neck-Odelberg Disease” and “Ischiopubic Osteochondrosis.” Manual citation searches and citation tracking were also performed to ensure inclusion of all relevant studies.

Selection Process

All search results from each database were imported into Covidence, a management software used to organize systematic review data, which automatically screened for duplicates [9]. Titles and abstracts were independently screened by two authors (CJG and RNC), who excluded irrelevant studies. Full-text articles were then independently screened for inclusion eligibility by two authors (CJG and RNC). A third author (MDM) mediated any conflicts in consensus between the first two article screeners (CJG and RNC).

Data Extraction and Items Collected

After the final set of articles was collected, data extraction was accomplished by two authors (CJG and RNC) and subsequently verified for completeness and correctness by a third author (MDM). Data extracted from each case report or case series included the last name of the first author, year of publication, article type, patient age (years), sex of the patient, patient activities, Extracted information on the clinical course consisted of (1) details of the initial presentation; (2) time to presentation or referral; (3) differential diagnoses; (4) laboratory values: erythrocyte sedimentation rate (ESR), C-reactive protein (CRP), white blood cell (WBC) count, and red blood cell (RBC) count; (4) diagnostic imaging: CT, MRI, and X-ray; (5) treatment regimens; and (6) information about symptom resolution as well as follow-up data.

Bias Assessments of Included Studies

The Joanna Briggs Institute (JBI) critical appraisal checklist for case reports and case series was used to dictate the quality of the included case reports and case series [10,11]. The JBI critical appraisal checklist for case reports consists of an eight-question checklist. This checklist was used to evaluate the comprehensiveness of the data included within each case report, including patient demographics, patient’s past medical history, presenting symptoms, diagnostic tests and assessment methods, interventions and treatments, post-intervention clinical condition, adverse or unanticipated events, and summarized takeaway lessons [10]. The JBI critical appraisal checklist for case series consists of a 10-question checklist. This checklist allowed for proper evaluated of the data included within each case series, including the inclusion criteria for the case series, the method of condition standardization for all patients included in the case series, condition identification methods, patient demographics and clinical information, outcomes and results of cases, clarity of reporting of presenting site or clinic demographic information, and whether statistical analysis was appropriate [11].

Results

Study Selection

**Figure 1:**
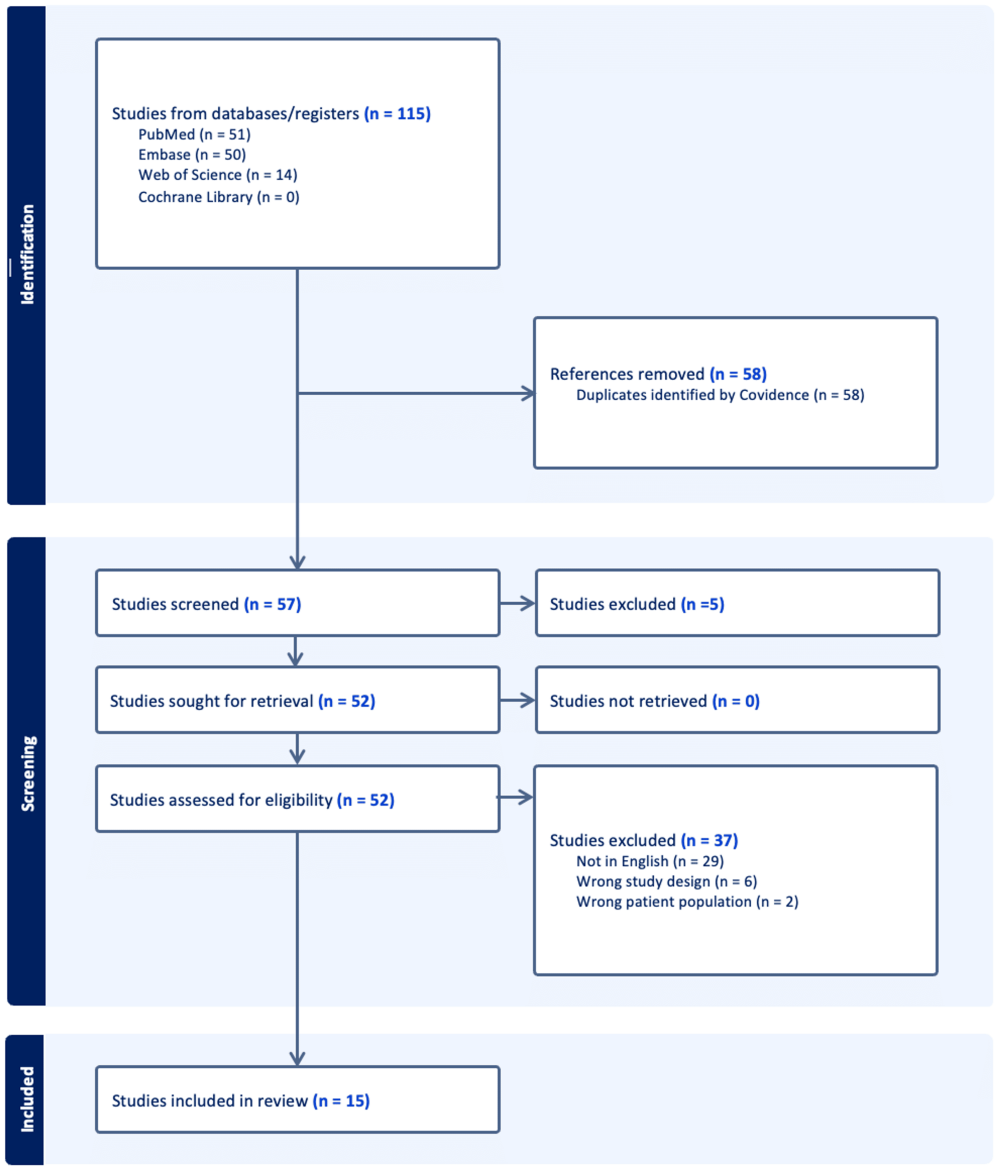
Preferred Reporting Items for Systematic Reviews and Meta-Analyses (PRISMA) flowchart detailing the literature search and data selection process.

In total, 115 articles were identified utilizing search terms in four databases: PubMed (n = 51), Embase (n = 50), Web of Science (n = 14), and Cochrane (n = 0). Covidence automatically removed 58 duplicate articles, leaving 115 articles to screen titles and abstracts for eligibility. Five studies were deemed irrelevant while performing a title and abstract screening, leaving 52 articles to be screened during a full-text review. Of these 52 articles, 37 were excluded (29 studies were excluded for not being written in English, six studies were excluded for having the wrong study design, and two studies were excluded for having the wrong patient population). The resulting 15 studies were included in this study (Figure 1).

Study Characteristics, Patient Characteristics, and Clinical Assessment of VND

The 15 studies included in this systematic review described 19 cases of VND. The baseline characteristics of each study, the study participants, and clinical course are shown in Table 1. Of the 19 cases, 16 were male (84.2%). The median age was 12 years (range = 5.5-17 years). The median time to presentation was 60 days (range = immediate presentation to 2 years). The most common presenting symptoms were hip and groin pain (n = 16; 84.2%), limping and gait limitation (n = 9; 47.4%), restricted hip range of motion (n = 4; 21.1%), a tender ischial tuberosity (n = 3; 15.8%), a tender IPS (n= 3; 15.8), and tender hip adductors (n = 2; 10.5%). Other presenting symptoms include inability to bear weight on the affected side (n = 1; 5.3%), dynamic flat foot (n = 1; 5.3%), bilateral valgus knees (n = 1; 5.3%), and reduced hip rotator and adductor strength (n = 1; 5.3%). The most common treatments were rest (n = 11; 57.9%), analgesics (n = 10; 52.6%), abstinence from sports (n = 6; 31.6%), conservative management (n = 6; 31.6%), partial weight bearing with crutches (n = 3; 15.8%), and one individual experienced symptom resolution with pain-adapted full weight bearing. The most common differential diagnoses were fractures (n = 7; 36.8%), tumor of the ischial ramus (n = 6; 31.6%), and osteomyelitis (n = 5; 26.3%). Other differentials included fibrous dysplasia (n = 1; 5.3%), post-traumatic osteolysis (n = 1; 5.3%), tuberculosis (n = 1; 5.3%), slipped capital femoral epiphysis (n = 1; 5.3%), eosinophilic granuloma (n = 1; 5.3%), bone cyst (n = 1; 5.3%), osteochondrosis (n = 1; 5.3%), infection (n = 1; 5.3%), and enthesitis-related arthritis (n = 1; 5.3%). The median time to resolution was eight weeks (range = 2 days to 3 years). Overall, 15 of the 19 individuals included in this study experienced complete resolution of symptoms.

**Table 1:** Patient characteristics extracted from all included studies. DDx = differential diagnosis; ROM = range of motion; NWB = non weight bearing; f/u = follow-up; NSAID = non-steroidal anti-inflammatory drug; IPS = ischiopubic synchondrosis; SCFE = slipped capital femoral epiphysis; PWB = partial weight bearing; FWB = full weight bearing; MRI = magnetic resonance imaging; hx = history; VND = Van Neck-Odelberg disease

Author (year)	Article type	Age (years)	Sex	Activities	Presenting symptoms	Time to presentation	DDx	Treatment plan	F/u and results
Beyitler and Kavukcu (2016) [13]	Case report	7	Male	-	Radiating groin pain; NWB on affected side; tender hip adductors; restricted hip ROM	Immediate presentation	Subacute fracture	Rest; analgesics	3-week f/u revealed complete resolution of symptoms
Camacho et al. (2020) [17]	Case report	15	Nale	-	Non-radiating, sharp pain in iliac fossa and hypogastrium; gait limitation	3 months	Ischiopubic ramus fracture	-	-
Ceri and Sperati (2020) [18]	Case report	8	Male	-	Groin pain to palpation; limping; restricted leg ROM	20 days	-	Rest; NSAIDs; abstain from sports until symptoms resolution	30-day f/u revealed complete resolution of symptoms
Ceroni et al. (2004) [19]	Case report	5.5	male	-	Gluteal pain, limping, no hx of trauma, tender IPS; no local swelling; contracture of adductor muscles; limited hip ROM	4 days	Osteomyelitis; tumor of the ischial ramus	Rest; NSAIDs	Complete resolution of symptoms within 48 hours
Chaudhari et al. (2017) [6]	Case report	12	Male	-	Groin pain; limping; no local swelling	2 months	Stress fracture; osteomyelitis; tuberculosis; post-traumatic osteolysis; neoplasia	Rest for 3 weeks; NSAIDs	3-month f/u revealed complete resolution of symptoms
Fonseca et al. (2022) [16]	Case report	6	Male	Futsal	Inguinal pain; limping; bilateral valgus knees; dynamic flat foot; tender ischiopubic area and adductors; limited hip ROM; reduced adductor and rotator muscle strength	-	-	Rest; abstain from sports for 6 weeks	8-week f/u revealed minimal improvement, continued pain, and a limp. A rehabilitation program was prescribed.
Laliotis et al. (2022) [14] patient 1	Case series	13	Male	Football	Hip pain; limping; normal ROM and mobility; tender ischium	3 weeks	SCFE	Rest; PWB with crutches for 1 month	3-month f/u revealed complete resolution of symptoms
Laliotis et al. (2022) [14] patient 2		14	Male	Sports	Tender ischial tuberosity	3 months		PWB with crutches for 1 month; abstain from sports	4-month f/u revealed complete resolution of symptoms
Laliotis et al. (2022) [14] patient 3		11	Male	sports	Tender ischial tuberosity	3 months		PWB with crutches for 1 month; abstain from sports	5-month f/u revealed complete resolution of symptoms
Laliotis et al. (2022) [14] patient 4		10	Female	Dancing	Hip pain	2 months	Subacute osteomyelitis	Conservative management	4-week f/u revealed complete resolution of symptoms
Macarini et al. (2011) [20] patient 1	Case series	8	Male		Ischiopubic pain; limping; no adductor muscle contracture	1 month		Rest; NSAIDs	3-week f/u showed drastic symptom improvement; 4-week f/u MRI revealed decreased perilesional edema
Macarini et al. (2011) [20] patient 2		12	Male	Football	Hip pain; inability to ambulate	Immediate presentation		Rest; NSAIDs	2-week f/u revealed complete resolution of symptoms
Mixa et al. (2017) [1]	Case report	15	Female		Groin pain	Immediate presentation	-	Conservative management	3-month f/u revealed complete resolution of symptoms
Morse and Lin (2016) [12]	Case report	17	Female	Soccer, cheerleading	Groin and thigh pain; no hx of trauma; mild pain in the ischial tuberosity region with resisted knee flexion	2 years	Acute hematogenous osteomyelitis; stress fracture; enchondroma; Ewings sarcoma or osteosarcoma; fibrous dysplasia; eosinophilic granuloma; bone cyst; osteochondrosis	Conservative; abstain from sports until symptom resolution	4-month f/u revealed worsening symptoms; 1-year f/u revealed complete resolution of symptoms
Pirimoglu and Sade (2019) [21]	Case report	8	Male	-	Groin pain; no hx of trauma	Immediate presentation	Fracture, infection, or bone tumor	Rest; NSAID for 3 weeks	-
Sabir et al. (2021) [5]	Case report	14	male	-	Gluteal, groin, and perineal pain; restricted physical activity; no hx of trauma	1 year	Stress fractures, osteomyelitis, neoplastic processes, and enthesitis-related arthritis	Rest; 3-week course of NSAIDs	3-year f/u revealed complete resolution of symptoms
Schneider et al. (2020) [22]	Case report	12	Male	Sports	Inguinal pain	3 months	Bone tumor	Conservative management	6-month follow-up revealed complete resolution of symptoms
Tam et al. (2021) [23]	Case report	12	Male	-	Bilateral groin pain; limping	18 months	VND	Conservative management	Symptoms resolved with skeletal maturity
Walter et al. (2020) [24]	Case report	10	Male	-	Radiating groin pain; no hx of trauma	4 months	Pathological pubic ramus fracture	Pain-adapted FWB	4-month f/u revealed no disease progression

Diagnostic Workup of VND

Laboratory values, CT findings, MRI findings, and X-ray findings of the cases included in this study are described in Table 2. Laboratory findings included elevated CRP (n = 4; 21.1%), elevated ESR (n = 3; 15.8%), and increased leukocytes (n = 1; 5.3%). CT findings included irregular IPS margins (n = 3; 15.8%), an enlarged ischiopubic ramus (n = 2; 10.5%), adjacent soft tissue edema (n = 1; 5.3%), focal increased uptake at the IPS (n = 1; 5.3%), and delayed IPS closure (n = 1; 5.3%). While there are currently no known pathognomonic CT findings for this disease, these are the most commonly reported.

**Table 2:** Laboratory values and diagnostic imaging results of all included studies. CT = computed tomography; MRI = magnetic resonance imaging; WNL = within normal limits; VND = Van Neck-Odelberg disease; CRP = C-reactive protein; ESR = erythrocyte sedimentation rate; IPS = ischiopubic synchondrosis; WBC = white blood cell; ASO = anti-streptolysin O

Author (year)	Laboratory values	CT findings	MRI findings	X-ray findings
Beyitler and Kavukcu (2016) [13]	Increased leukocytes; normal CRP and ESR	-	Hypointense sclerotic discontinuity of anterior pubic ramus; perilesional edema	Enlarged right IPS with osteolytic areas
Camacho et al. (2020) [17]	WNL	Focal increased uptake at IPS; expansion of medial ischiopubic ramus; adjacent soft tissue edema	-	-
Ceri and Sperati (2020) [18]	WNL	-	VND of the left ischiopubic ramus	-
Ceroni et al. (2004) [19]	Elevated CRP and ESR; blood exam normal	-	Bone marrow of the right IPS as well as adjacent muscles had a hyperintense signal alteration; hypointense band centered the right ischiopubic bone marrow	Anteroposterior radiographs of pelvis revealed a normal appearance of the IPS; right-sided radiolucent swelling noted
Chaudhari et al. (2017) [6]	WBC WNL; ESR raised with positive CRP ratio	Enlarged non-fused IPS containing lytic lesions	Hyperintense signal on T2; hypointense signal on T1	Sclerotic shadow seen in IPS extending towards the obturator foramen
Fonseca et al. (2022) [16]	WNL	-	-	Demineralization and hypertrophy of the right IPS
Laliotis et al. (2022) [14] patient 1	-	-	Calcified osteochondral mass	Irregular calcification of right ischium
Laliotis et al. (2022) [14] patient 2	-	-	Edema of the ischium	Irregular cortex of the left ischium; line of calcification
Laliotis et al. (2022) [14] patient 3	-	-	Bilateral edema of the IPS	Enlargement and diastasis of the IPS
Laliotis et al. (2022) [14] patient 4	WNL	-	Ischial edema	Diastasis and enlargement of the IPS
Macarini et al. (2011) [20] patient 1	Increased CRP and ESR; normal ASO titer	Demineralization and sclerosis at edges of the synchondrosis	Hypointense band perpendicular to pubic axis; edema of tissue and muscles surrounding lesions	Right IPS was enlarged with osteolytic regions
Macarini et al. (2011) [20] patient 2	Increased CRP; normal ASO titer	Left IPS shows delayed closure and irregular bone margins	Left IPS fusiform enlargement	Left IPS enlargement with radiolucency
Mixa et al. (2017) [1]	-	-	Completed	Completed
Morse and Lin (2016) [12]	WNL	Calcifications within the lesion; cortices appeared focally incomplete anteriorly and posteriorly; areas of endosteal scalloping	Lesion had relatively low signal intensity on T1-weighted images but mirrored that of red marrow making it hyperintense to muscle	Well-marginated expansile lesion of the left inferior pubic ramus
Pirimoglu and Sade (2019) [21]	-	-	Hyperintense edema, swelling, corticomedullary discontinuity and slightly contrast enhancement pattern at left IPS	Enlarged left IPS characterized by focal osteolytic area
Sabir et al. (2021) [5]	WNL	-	Transverse hypointense fibrous band surrounded with edema in the region of the right IPS seen at low signal intensity on T1-weighted MRI	Expansion of the right IPS; epiphyses were open
Schneider et al. (2020) [22]	-	-	-	Extensive, partly demineralized geographic lesion with no periosteal reaction at the junction of the pubic bone and the ischium
Tam et al. (2021) [23]	-	-	Left fusiform IPS expansion with increased signal intensity in the bone and adjacent soft tissue; and absence of synovitis, soft tissue mass, or collections	Expansion of IPS bilaterally (left > right) with normal hips
Walter et al. (2020) [24]	-	-	Completed	Completed

Risk of Bias Assessment

The risk of bias assessment was assessed for all 15 included studies (2 case series and 13 case reports) using the JBI critical appraisal tool. One case report received “yes” to only six of the eight questions found on the JBI critical appraisal tool, while the remaining 12 case reports had a score of either 7 or 8. The average risk bias assessment score for all 13 included case reports was 7.15/8. Both case series included in this study received a JBI critical appraisal tool score of 9.

Discussion

Ischiopubic osteochondrosis is a cause of pediatric hip pain that is often misdiagnosed due to its rarity. Delaying proper diagnosis causes prolonged pain and impaired movement for individuals suffering from this disease, who are pediatric patients and often are unable to participate in activities, such as sports, until this disease is fully resolved.

Analysis of all published cases of VND reveals an interesting correlation between time until full symptom resolution and time to treatment from the initial onset of symptoms. While most patients received treatment upon presentation for the immediate onset of symptoms, some patients waited months or years before seeking professional help from clinicians. One case described a patient who waited two years after the initial onset of symptoms to see a pediatric orthopedic specialist and received a formal diagnosis of VND [19]. This patient was also the oldest of all published cases of VND (17 years old) and had one of the longest times to complete recovery (one year). This patient’s four-month follow-up suggested that the lesion had grown on subsequent imaging studies, but the study did not note any changes in her symptoms. At the sixth-month follow-up, her symptoms were improving, and she progressed in her activities. A subsequent follow-up at one year, the patient was asymptomatic and made a full return to athletic activities, along with the disappearance of the lesion on imaging studies. Another patient presented one year after the onset of symptoms and saw a prolonged time to full symptom resolution (three years) [5]. There could also be a correlation between age at symptom onset and time to symptom resolution based on a review of all cases included in this study. There is a general pattern of older individuals having a prolonged time to full symptom resolution. The two aforementioned individuals are among the oldest included in this study, at 14 and 17 years of age. These individuals experienced the longest time to recovery of any individuals included in this study. Although patients older than 12 years old did have a longer time from the initial onset of symptoms to diagnosis and treatment of VND. This may be attributed to VND being lower on the differential diagnosis in this age group and the rarity of the condition itself compared to other conditions on the differential diagnosis. In the oldest reported patient, a 17-year-old female, the presentation was unusual in that the pain only occurred after activity such as playing soccer or cheerleading and resolved after 24-48 hours.

One issue commonly encountered with individuals enduring VND pain is an incorrect initial diagnosis. These misdiagnoses result in delayed care, extending the duration of pain suffered by children with VND. VND frequently presents similarly and initially may be misinterpreted as other pathologies, including stress fractures, bone tumors, osteomyelitis, and post-traumatic osteolysis [12]. In this systematic review of the literature, 11 of the 19 patients were initially misdiagnosed with a wide array of pathologies, as previously mentioned, with stress, subacute, or pathological fractures being the most common. Stress fractures can be differentiated on MRI, which shows an irregular fracture line [19]. Avulsion fractures occur acutely during activity and present as pain and difficulty jumping and displacement of the fracture and muscle edema on MRI [17]. Additional differential diagnoses included slipped capital femoral epiphysis, fibrous dysplasia, eosinophilic granulomas, and bone cysts. Malignant neoplasms would show bone marrow, irregular bone cortex, and surrounding tissue edema early and abscess formation on MRI as a late finding in osteosarcoma or Ewing’s sarcoma. Enthesitis-related juvenile idiopathic arthritis would present as enthesitis and arthritis, along with sacroiliac pain, positive laboratory testing, other autoimmune conditions, or a family history of autoimmune conditions [5]. Two patients were given the diagnosis of VND and were not initially misdiagnosed, while there were six without any listed differentials mentioned on presentation.

An additional consideration for the resolution of VND in the pediatric population is the activity levels of the patient with VND before diagnosis. Research shows adequate activity levels are important not only for managing chronic diseases, preventing disease, and preventing premature death, but activity is extremely important for overcoming acute illness [24]. Of the patients included in this study, eight were athletes and participated in activities, including futsal, football, dancing, soccer, and cheerleading. Of these eight, six were found to have complete resolution of symptoms upon their initial follow-up visit. One athlete at eight weeks had minimal improvement, and with prescribed rehabilitation, had improvement of symptoms [16]. The other patient was found to have an increasing size of their lesion, and with continued therapy and rehabilitation, was able to succeed in activities of daily living at six months and returned to sporting events after one year [19]. Comparing athletes versus non-athletes, there was no uniform follow-up time among patients. Follow-up times ranged from 48 hours and two weeks on the shorter end up to six months.

While most patients achieved full recovery after initial treatment, one case involved a six-year-old boy diagnosed with VND and treated with relative rest, suspension of sports practice for six weeks, moderation in running activities, and follow-up at eight weeks [16]. The patient, despite recommendations, resumed futsal practice two weeks after the diagnosis with no change in training intensity. This led to symptom recurrence and impairment. On follow-up, the patient still reported groin pain and a slight limp, and a rehabilitation program was recommended. Following rehabilitation, re-evaluation after six months showed a complete resolution of symptoms and gait improvement.

Limitations

This study was not without limitations. In this systematic review, only English-language publications were included. Therefore, some of the earlier texts regarding this disease process were excluded. Additionally, the data provided across all studies were not homogeneous. However, all available data were extracted from the studies to provide readers with the most comprehensive overview of VND to date. Lastly, due to the limitation of including English-only studies, there may be several relevant cases omitted, particularly from Europe or Asia.

## Conclusions

VND, or IPS, is a rare and difficult-to-diagnose pediatric condition due to its nonspecific presentation. Common differential diagnoses include fractures, osteomyelitis, benign or malignant tumors, slipped capital femoral epiphysis, and autoimmune disorders. Recognizing the clinical characteristics of VND, such as its associated imaging, physical examination findings, and laboratory findings, can help make an accurate diagnosis and prevent delayed treatments. It is particularly important when conducting the clinical work-up for older pediatric individuals to always keep VND in the differential when similar nonspecific symptoms are present to prevent longer recovery times in this demographic. Even though this pathology is treated with conservative management and typically has a good prognosis, making the correct initial diagnosis is imperative to preventing unnecessary invasive management. Understanding the anatomy and physiology of this normal, temporary joint and being able to recognize common diagnostic features will allow physicians to accurately identify this disease.
